# Anabolic Drugs and Myocardial Infarction – A Clinical Case Report

**DOI:** 10.5935/abc.20150111

**Published:** 2015-09

**Authors:** Rui Pontes Santos, Adriana Pereira, Henrique Guedes, Carolina Lourenço, João Azevedo, Paula Pinto

**Affiliations:** Serviço de Cardiologia, Centro Hospitalar Tâmega e Sousa, Penafiel – Portugal

**Keywords:** Anabolic Agents/adverse effects, Human Growth Hormone, Myocardial Infarction, Heart Failure, Clembuterol, Weight Lifting

## Introduction

In most cases of myocardial infarction (MI) in young people, traditional cardiovascular risk factors continue to be of utmost importance; but at this age, other causes should also be considered, such as drug abuse.

Anabolic steroids are synthetic derivatives of testosterone and are often illegally used by athletes to increase their physical performance. There is a correlation between the use of these drugs and increased cardiovascular risk^1^. Human growth hormone (hGH) has also been used to increase physical performance, despite the risk of cardiovascular complications^2^. Clenbuterol is a potent β2-agonist that also has anabolic effects. However, at present, little is known about its potential cardiovascular risk^3^.

Despite reports of MI associated with the use of anabolic steroids, no previous studies have reported the simultaneous use of these three types of medications.

## Case Report

A 25-year-old Caucasian male had no cardiovascular risk factors or history of other known relevant diseases. His practiced bodybuilding and regularly consumed anabolic steroids, hGH, and clenbuterol for the past 6 months. The last cycle, initiated 6 weeks before the episode described herein, comprised the following: oxandrolone, 40 mg/day (daily); clenbuterol, 0.08 mg/day (daily); mesterolone, 50 mg/day (daily); hGH, 10 IU/day (daily); nandrolone, 600 mg/day (twice a week); testosterone cypionate, 400 mg/day (twice a week); stanozolol, 100 mg/day (thrice a week); drostanolone, 200 mg/day (thrice a week); trenbolone at 200 mg/day (thrice a week); testosterone propionate, 100 mg/day (thrice a week); boldenone, 400 mg/day (twice a week); and methenolone, 200 mg/day (twice a week). The patient denied having smoked or used any other drugs.

The patient complained of intense oppressive retrosternal pain , without irradiation or other accompanying symptoms; the pain lasted approximately 2 hours and was associated with muscle fatigue after training. Approximately 24 hours after the initial episode, the patient experienced recurrence of the pain, which worsened with inspiration (different from the initial pain); he was therefore referred to emergency care.

**Figure 1:**
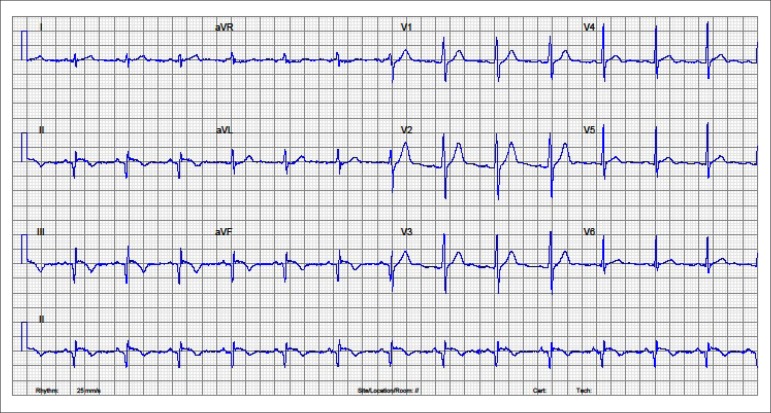
Electrocardiogram on admission with signs of infarction on the inferior and posterior left ventricular walls in the subacute phase.

Upon admission to the emergency department, he was asymptomatic and physical examination indicated hemodynamic stability and fever (38.4ºC) with no other significant findings. The electrocardiogram (ECG) showed sinus rhythm, heart rate of 83 beats/minute, with signs of infarction on the inferior and posterior walls of the left ventricle (LV) in the subacute phase (Figure 1). Laboratory tests showed increased levels of the following myocardial necrosis markers: CPK, 1987 IU/L (reference value [RF] < 172 IU/L); troponin I, 48.97 ng/mL (RF < 0.05 ng/mL) on admission, with subsequent tendency to decrease; BNP, 115 pg/mL (RF < 100 pg/mL); and CRP, 33.3 mg/L (RF < 7.5 mg/L), with a maximum peak of 204.4 mg/L on admission. The clotting factors were within normal limits. The patient was started on double platelet antiaggregant therapy with acetylsalicylic acid and clopidogrel and anticoagulation therapy with fondaparinux.

He was admitted to the cardiac intensive care unit with a diagnosis of MI with ST-segment elevation of posteroinferior location in the subacute phase, of Killip Kimball class I, associated with likely post-infarction pericarditis. Echocardiographic examination indicated hypokinesia of the middle and basal segments of the inferior, posterior, and lateral LV walls. Of note was the presence of mild concentric LV hypertrophy and the preservation of the overall biventricular systolic function.

**Figure 2:**
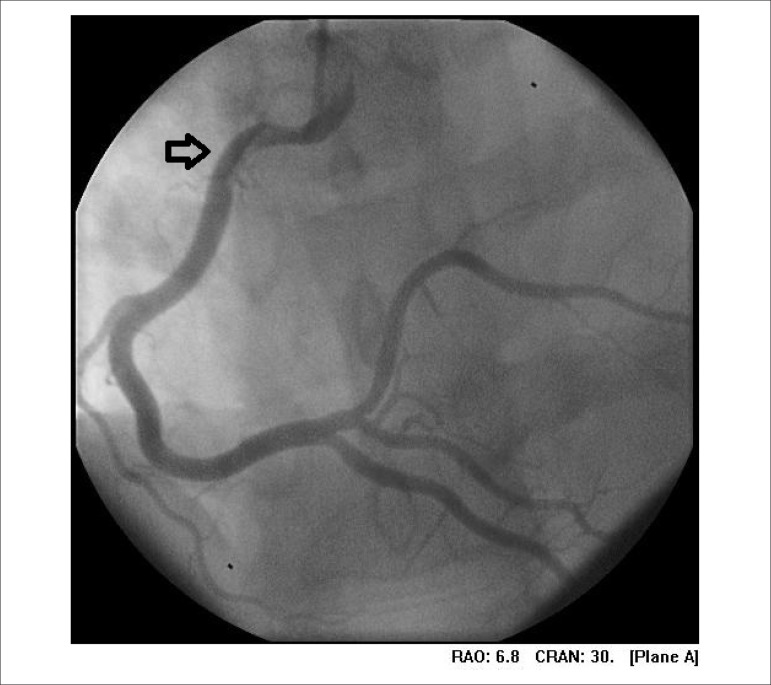
Coronary angiography examination indicated intraluminal thrombus in the proximal third of the right coronary artery.

Approximately 48 hours after the onset of symptoms, the patient underwent coronary angiography, which indicated fusiform stenosis in the proximal third of the right coronary artery, suggestive of intraluminal thrombus, but it did not represent a significant stenosis condition (Figure 2). He showed no changes suggestive of atherosclerotic epicardial coronary disease. Owing to the small size of the thrombus, it was decided to continue the anticoagulation therapy until the end of hospitalization and prolong the double antiaggregant therapy with the use of maintenance doses.

During hospitalization, he presented a favorable outcome and remained electrically and hemodynamically stable, without pain recurrence and with analytical improvement. Being asymptomatic, he was discharged on the eighth day of hospitalization, with a prescription of aspirin (150 mg od), clopidogrel (75 mg od), bisoprolol (5 mg od), ramipril (2.5 mg od), and rosuvastatin (10 mg od).

He initiated follow-up with a cardiology consultation. One year after hospital discharge, he remained abstinent from anabolic substances and was asymptomatic from the cardiovascular point of view. ECG and echocardiography examination performed at the last follow-up maintained the changes described previously. In addition, a stress test was conducted, which indicated no changes suggestive of MI.

## Discussion

Although uncommon, some MI cases have been reported among young individuals without cardiovascular risk factors and who use anabolic steroids^4-7^. Although this cause-effect relationship is not fully understood, some hypotheses have been proposed to explain the cardiovascular adverse effects of anabolic steroids^8^.

Some studies attribute a thrombotic effect to anabolic steroids. These drugs appear to be associated with increased platelet aggregation, as a result of the increased production of thromboxane A2 and decreased production of prostacyclin. In addition, changes in the coagulation cascade may occur, including increased thrombin activity, which also contributes to a hypercoagulable state^1^. These adverse effects are exacerbated by dehydration and catecholaminergic stress, which often occur in association with physical activities^8^.

hGH has also been associated with cardiovascular complications. hGH abuse contributes to increased heart rate and cardiac output, consequently leading to concentric ventricular hypertrophy and diastolic dysfunction; in certain patients, it may even promote ischemia/necrosis and heart failure associated with impairment of the systolic function^2^.

Clenbuterol, when administered orally, appears to have anabolic effects, contributing to an increase in muscle mass^3^. Despite limited information about the potential cardiac complications, two cases of MI have been reported in which clenbuterol may have had a key role^9-10^. It is speculated that its positive chronotropic and inotropic effects, in addition to the redistribution of coronary circulation, may promote myocardial ischemia^8^.

Therefore, we believe that this patient suffered from an MI due to anabolic steroid abuse and that it was a type 2 MI, in which intraluminal thrombus formation occurred due to a hypercoagulable state associated with the use of anabolic steroids. In this case, it is important to stress the possible synergistic effect of the concomitant use of hGH and clenbuterol. It has been shown that both drugs promote myocardial ischemia, which allows us to hypothesize that all three drugs may have contributed to the final clinical outcome of this patient.

In conclusion, the use of anabolic steroids seems to be a risk factor for the development of acute coronary syndrome. This reinforces the idea that it is necessary to exclude a previous history of consumption of illicit drugs in the presence of an MI, particularly in patients with low cardiovascular risk.
